# The role of iron in anthracycline cardiotoxicity

**DOI:** 10.3389/fphar.2014.00025

**Published:** 2014-02-26

**Authors:** Elena Gammella, Federica Maccarinelli, Paolo Buratti, Stefania Recalcati, Gaetano Cairo

**Affiliations:** ^1^Department of Biomedical Sciences for Health, University of MilanoMilano, Italy; ^2^Department of Molecular and Translational Medicine, University of BresciaBrescia, Italy

**Keywords:** iron, anthracyclines, heart, doxorubicin, reactive oxygen species

## Abstract

The clinical use of the antitumor anthracycline Doxorubicin is limited by the risk of severe cardiotoxicity. The mechanisms underlying anthracycline-dependent cardiotoxicity are multiple and remain uncompletely understood, but many observations indicate that interactions with cellular iron metabolism are important. Convincing evidence showing that iron plays a role in Doxorubicin cardiotoxicity is provided by the protecting efficacy of iron chelation in patients and experimental models, and studies showing that iron overload exacerbates the cardiotoxic effects of the drug, but the underlying molecular mechanisms remain to be completely characterized. Since anthracyclines generate reactive oxygen species, increased iron-catalyzed formation of free radicals appears an obvious explanation for the aggravating role of iron in Doxorubicin cardiotoxicity, but antioxidants did not offer protection in clinical settings. Moreover, how the interaction between reactive oxygen species and iron damages heart cells exposed to Doxorubicin is still unclear. This review discusses the pathogenic role of the disruption of iron homeostasis in Doxorubicin-mediated cardiotoxicity in the context of current and future pharmacologic approaches to cardioprotection.

## INTRODUCTION

Doxorubicin (DOX) is a widely used anthracycline anticancer drug that intercalates in DNA and inhibits topoisomerase II. More than four decades after discovery, DOX still ranks among the most effective agents in breast cancer and numerous other neoplastic diseases. Unfortunately, the clinical use of DOX is limited by the possible development of cardiomyopathy and congestive heart failure (CHF). The various forms of anthracycline-mediated cardiotoxicity, as well as the reasons for the particular susceptibility of cardiac cells, have been reviewed elsewhere (Minotti et al., 2004a; Berthiaume and Wallace, 2007; Octavia et al., 2012; Carvalho et al., 2014); here we will summarize the major clinical consequences of this toxicity, and then review the biochemical mechanisms of anthracycline cardiotoxicity, focusing on the role of iron.

Since a tight relationship between the quantity of DOX accumulated in the heart and the incidence of cardiac events has been identified, the lifetime cumulative dose of DOX usually does not exceed 500 mg/m^2^; this reduces considerably the incidence of treatment-requiring cardiac events, but sometimes can preclude successful completion of chemotherapy. Moreover, cardiomyopathy may develop at lower doses in the presence of risk factors like age, hypertension, arrhythmias, coronary disease, etc. (Gianni et al., 2008). Therefore, the dose-adjusted chance of cardiotoxicity may be more elevated than expected, and the necessity to adjust doses limits the therapeutic capability of DOX and other anthracyclines. It should also be considered that anthracycline cardiotoxicity is often exacerbated by combination therapies involving classic drugs like taxanes or new drugs targeting growth factor receptors or signal transducing molecules activated in tumors (Gianni et al., 2008).

Since at present there is no specific and effective pharmacological treatment for DOX-related cardiomyopathy, cardiac transplantation often remains the only option for patients with DOX-dependent advanced cardiomyopathy and CHF. It is therefore evident that the risk of developing cardiac events because of anthracycline treatment is a life-threatening problem for a high number of cancer survivors. In particular, given the peculiar delay in the appearance of toxic consequences, which may become manifest several years after completing chemotherapy (Minotti et al., 2004a), DOX cardiotoxicity represents a serious problem in pediatric oncology (Lipshultz et al., 2013a).

Despite convincing evidence linking the levels of DOX in the heart and the development of cardiomyopathy, the precise mechanisms through which DOX eventually induces cardiac damage are much less clear; this uncertainty is also dependent on obstacles to investigation posed by species-related differences in anthracycline metabolism and susceptibility to DOX-derived reactive oxygen species (ROS; see below), as well as to inherent difficulties in developing animal models reliably recapitulating chronic and delayed cardiotoxicity.

**FIGURE 1:**
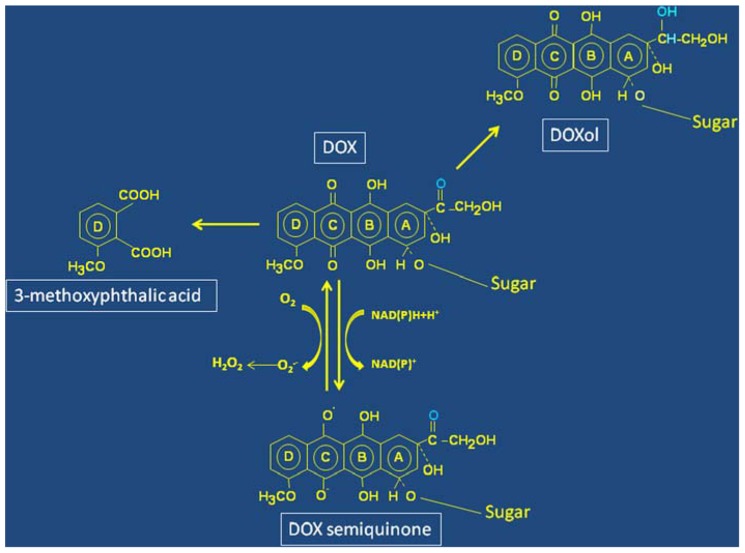
**Structure and simplified scheme of molecular transformations of Doxorubicin.** Redox cycling between the quinone and semiquinone forms (ring C) of Doxorubicin (DOX) leads to oxygen radicals formation. The residue involved in DOXol formation following two-electron reduction of the carbonyl group in ring A is marked in blue. Oxidative pathways involving a hydroquinone-derived semiquinone lead to formation of a diquinone (rings B and C), and eventual degradation of DOX with formation of 3-methoxytphthalic acid as remnant of ring D.

Anthracycline-induced cardiotoxicity is a multifactorial process based on mechanisms only in part distinct from those targeting cancer cells. In fact, it has been demonstrated that the interaction of DOX with topoisomerase, which is key for the antitumor effect of anthracyclines, is also relevant for cardiotoxicity (Zhang et al., 2012). Other mechanisms include suppression of the cardiac-specific gene expression program, persistent alterations of calcium compartmentalization, changes in adrenergic function, inhibition of the expression of several sarcomeric proteins, promotion of free radical reactions beyond the limited antioxidant defence of cardiomyocytes that eventually lead to lipid peroxidation (Minotti et al., 2004a). Great attention is paid to the latter mechanism because a number of studies have shown that reductive activation of the quinone moiety of DOX (**Figure 1**) by a number of reductases, including the reductase domain of endothelial nitric oxide synthase (Fogli et al., 2004), eventually results in the formation of superoxide anion (O_2_^•-^) and hydrogen peroxide (H_2_O_2_) that may cause oxidative stress and cell injury in cardiomyocytes (Minotti et al., 2004b; Berthiaume and Wallace, 2007; Octavia et al., 2012; Stěrba et al., 2013; Carvalho et al., 2014). In this context, the role of ROS-generating mitochondrial quinone reductases appears particularly relevant, as also indicated by the abundance of mitochondria in the heart. In addition to biochemical evidence, the role of ROS produced by the reductases was underlined by studies in genetically modified mice showing a relation between cardiotoxicity and either deletion of enzymes involved in anthracycline metabolism or overexpression of antioxidant proteins (reviewed in Minotti et al., 2004a). Interestingly, a high-risk variant of a gene involved in free radical generation significantly increased the susceptibility to DOX-dependent CHF in survivors of hematopoietic cell transplantation (Armenian et al., 2013).

Other pathways of DOX metabolism are also highly relevant for cardiotoxicity. Reduction of the carbonyl group in ring A to alcohol (**Figure 1**) changes DOX into DOXol that, being more polar than DOX, accumulates at higher levels and for longer times in the heart, thus helping to understand chronic cardiotoxicity. Moreover, it has been shown that DOXol is remarkably more potent than its parent molecule at dysregulating iron homeostasis (see below). The role of DOXol in cardiotoxicity is reinforced by studies showing correlation between polymorphisms in the genes coding for carbonyl reductases that form DOXol and cardiovascular morbidity (Blanco et al., 2008).

However, in addition to the reductive metabolism described above, DOX can undergo oxidative degradation catalyzed by peroxidases. Recent elegant studies identified H_2_O_2_-activated myoglobin as the catalyst of this reaction, and 3-methoxyphthalic acid, the final product of DOX oxidative degradation (**Figure 1**) was detected in hearts of mice exposed to DOX or human heart stripes incubated with DOX (Menna et al., 2010). Importantly, 3-methoxyphthalic acid proved to be much less toxic to cultured cardiomyocytes and mice than equimolar amounts of DOX, possibly because this metabolic pathway prevents quinone-semiquinone redox cycling. These findings suggest that anthracycline oxidative degradation may represent a salvage pathway that diminishes DOX cardiotoxicity (Menna et al., 2010).

## IRON AND ANTHRACYCLINE CARDIOTOXICITY

The relative weight of each individual mechanism in the development of anthracycline-induced cardiotoxicity is uncertain, but the role of iron in DOX cardiotoxicity is widely recognized (Minotti et al., 1999; Xu et al., 2005; Simůek et al., 2009).

Perhaps the most compelling proof is provided by the effectiveness of the iron chelator dexrazoxane at preventing cardiotoxicity, both in animal and clinical studies. The use of dexrazoxane in patients has been debated after reports showing myelotoxicity and interference with anticancer effects of DOX, and others with opposite conclusions (discussed by Stěrba et al., 2013). Nevertheless, the observation that dexrazoxane protects cancer patients against cardiotoxicity demonstrates that alterations in iron homeostasis are involved in anthracycline-induced CHF.

Additional and complementary experimental evidence for the role of iron in DOX cardiotoxicity was provided by studies showing that iron overload increases the cardiotoxic effects of the drug. Early studies showed that iron loading exacerbated DOX toxicity in cell culture (Hershko et al., 1993; Link et al., 1996). Moreover, increasing iron stores in rats by dietary iron loading enhanced DOX cardiotoxicity (Panjrath et al., 2007), and mice lacking HFE, a model that mimics the iron overload found in human hereditary hemochromatosis, showed higher susceptibility to DOX-dependent cardiac damage (Miranda et al., 2003). Importantly, a recent study showed that DOX-induced myocardial injury was associated with the frequency of HFE gene mutations in survivors of childhood acute lymphoblastic leukemia (Lipshultz et al., 2013b).

## IRON-DEPENDENT MECHANISMS OF ANTHRACYCLINE CARDIOTOXICITY

It is considered that by forming anthracycline-iron complexes iron potentiates the toxicity of DOX-derived ROS transforming relatively safe species like O_2_^•-^ and H_2_O_2_ into the much more reactive and toxic hydroxyl radical OH^•^ or iron-peroxo complexes that damage DNA, proteins, and lipids (Minotti et al., 1999; Xu et al., 2005; Simůnek et al., 2009). Moreover, it has been proposed that the redox cycling of the quinone moiety (**Figure 1**) would enable anthracyclines to increase the cellular levels of iron by mobilizing it from ferritin (see below), and thus amplify iron-mediated oxidative stress. Indeed, several lines of evidence in preclinical models indicated that iron and oxygen radicals conspire at inducing cardiotoxicity. However, the so-called “ROS and iron hypothesis” has been called into question; in fact, while iron chelators like dexrazoxane mitigate anthracycline cardiotoxicity, none of several antioxidants proved to be protective against chronic cardiotoxicity in clinical settings (Minotti et al., 2004a; Octavia et al., 2012; Stěrba et al., 2013).

Accordingly, we showed that DOX doses lower than those usually adopted in experimental studies, but in the range of the plasma levels found in patients undergoing slow infusion chemotherapy induced apoptosis of cardiac-derived H9c2 myocytes in the absence of ROS production, thus supporting the idea that oxidative stress does not play a role in DOX-dependent apoptotic death of cardiomyocytes (Bernuzzi et al., 2009).

We recently provided evidence suggesting an alternative mechanism at the basis of the protective capacity of iron chelators against cardiotoxicity (Spagnuolo et al., 2011). Treatment with dexrazoxane induced hypoxia inducible factor (HIF) in H9c2 cardiomyocytes. Moreover, it prevented the activation of apoptosis caused by treatment of H9c2 cells with low but pharmacologically relevant doses of DOX. The role of HIF was demonstrated by experiments involving pharmacological or genetic manipulation of HIF activity: the inhibition of HIF activity blunted the protective effect of iron chelation. Conversely, overexpressing HIF conferred protection from DOX-dependent cell death also in the absence of dexrazoxane. HIF exerted its defensive effect by activating the expression of protective proteins, as treatment with the chelator induced the synthesis of anti-apoptotic proteins known to be HIF targets.

These results suggest that the HIF pathway may contribute to the cardioprotective effect of iron chelation. Moreover, these results reinforce the idea that the protection against DOX toxicity provided by the iron chelator may be ROS-independent.

## EFFECT OF DOXORUBICIN ON PROTEINS OF IRON METABOLISM

**FIGURE 2:**
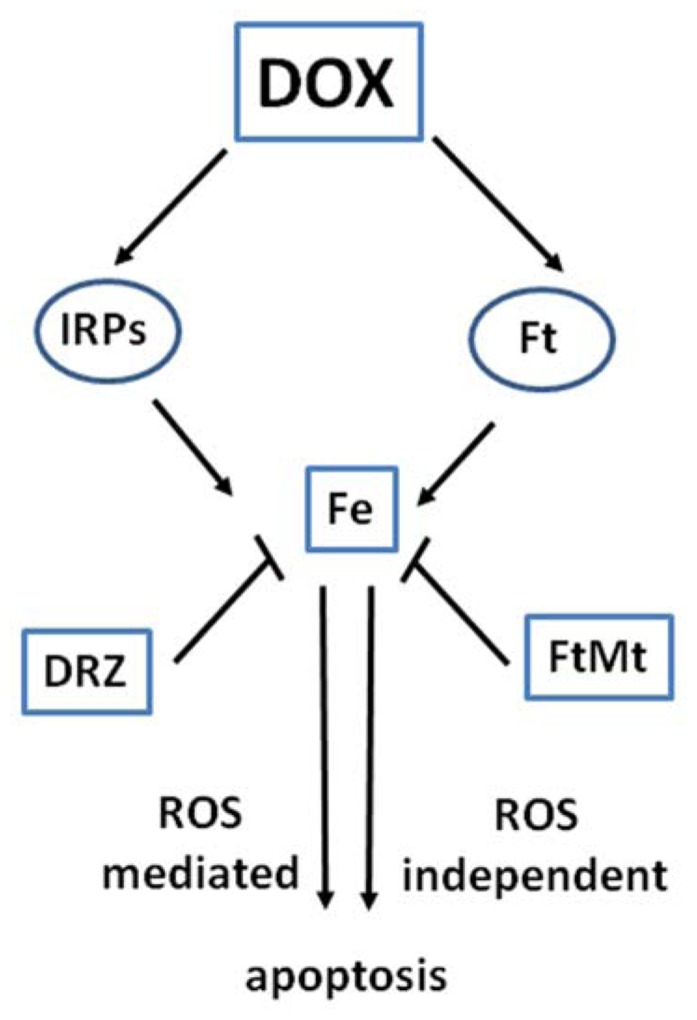
**Interaction of DOX with proteins of iron metabolism.** The interaction of DOX with the functions of iron regulatory proteins (IRPs) and ferritin (Ft) affects iron homeostasis and may lead to ROS-dependent and independent damage and apoptotic cell death. Iron sequestration by the iron chelator dexrazoxane (DRZ) or mitochondrial ferritin FtMt may prove cardioprotective.

The data reported above, together with evidence discussed below, indicate that the role of iron in DOX cardiotoxicity may be mediated not by DOX–iron interactions, but by profound interference with proteins involved in intracellular iron traffic (**Figure 2**). This concept is in agreement with the essential role of iron in regulating fundamental cellular processes like DNA synthesis, energy production, calcium channel function, redox balance, etc. (Hentze et al., 2004).

### IRON REGULATORY PROTEINS

The relationship between iron and anthracycline cardiotoxicity may be related to disruption of cardiac iron homeostasis obtained by means of targeted interaction of DOX with iron regulatory proteins (IRP1 and IRP2), the key regulators of intracellular iron metabolism (Cairo and Pietrangelo, 2000; Rouault, 2006; Cairo and Recalcati, 2007; Muckenthaler et al., 2008). IRPs form the basis of a homeostatic mechanism that optimizes the metabolic utilization of iron while avoiding its involvement in potentially toxic reactions. In mammalian cells, iron levels are finely controlled by means of opposite but balanced regulation of ferritin, the iron sequestering protein, and transferrin receptor (TfR1), the major iron uptake protein (Cairo and Pietrangelo, 2000). The post-transcriptional control of the cellular levels of ferritin and TfR1 is exerted by IRP-1 and IRP-2, cytosolic proteins that bind to iron regulatory elements (IRE) in ferritin and TfR1 mRNAs (Recalcati et al., 2010). Under conditions of iron scarcity, binding of IRPs to IRE represses the translation of ferritin mRNAs and at the same time enhances the half-life of TfR1 mRNA. This results in increased iron availability for the requirements of the cell. On the contrary, when iron is abundant, IRP activity is decreased, and thereby ferritin mRNAs are translated whereas TfR1 mRNA is degraded; hence, this mechanism facilitates iron storage. It is noteworthy that IRPs can regulate mRNAs for other proteins closely related to iron utilization (mitochondrial aconitase and δ-aminolevulinic acid synthase), uptake (DMT1), and release (ferroportin). Hence, their influence extends over a number of regulatory pathways controlling the iron status of the cell (Cairo and Recalcati, 2007).

IRP1 is a bifunctional protein that is also able to act as an aconitase, thus converting citrate to isocitrate, when it contains a [4Fe–4S] cluster (i.e., in iron-rich cells). On the other hand, when intracellular iron levels are low and the cluster is not formed, IRP-1 becomes an RNA-binding protein. Switching between these two forms therefore represents a mechanism allowing aconitase/IRP1 to adapt iron trafficking to the cell requirements. By providing isocitrate to isocitrate dehydrogenase, IRP-1 may also control the cellular levels of NADH+ H^+^. IRP2, which is similar to IRP1 but cannot assemble a [4Fe–4S] cluster, is ubiquitinated and degraded by the proteasome in response to iron, whereas it builds up in iron poor cells. IRP-2 degradation also occurs following oxidative modifications induced by reactive oxygen and nitrogen intermediates (Cairo and Pietrangelo, 2000; Rouault, 2006; Cairo and Recalcati, 2007; Muckenthaler et al., 2008).

How DOX affects IRPs ? Pathways linking interactions of IRPs with DOX, DOXol, and quinone-derived ROS, have been characterized, and shown to play a role in cardiotoxicity (Cairo et al., 2002). Observations conducted on cell-free systems or cardiac cell lines have demonstrated that after interaction with DOXol IRP-1 first looses the [4Fe–4S] cluster and then is converted by ROS into a “null” protein lacking both RNA binding and enzymatic activities (Minotti et al., 1998, 2001). Other studies provided data indicating a slightly different mechanism, by which such a “null” protein would be formed by DOX–iron complexes (Kwok and Richardson, 2002). Moreover, ROS-mediated damage targets IRP-2 to proteolysis (Minotti et al., 1998, 2001). The attack of DOX, and in particular of its secondary alcohol metabolite, to IRPs may dysregulate iron homeostasis in the cell not only by disrupting IRP’s regulatory function, but also through the release of iron atoms from the Fe–S cluster of IRP1; this, rather than ferritin (see below), may represent a source of iron that facilitates iron-mediated free radical formation and apoptosis. The demonstration that anthracyclines can interact with IRE regions of mRNAs (Canzoneri and Oyelere, 2008), thus affecting IRP-mediated regulation of several proteins of iron metabolism, has indicated another possible pathway through which DOX can disrupt iron balance.

What could be the pathologic consequences of a DOX-dependent inactivation of both IRPs? It should be kept in mind that only mice lacking both IRPs are not viable; this indicates that one IRP is sufficient to maintain iron balance, even though IRP-1-deficient mice did not show any phenotype whereas mice lacking IRP-2 have neurological and hematological abnormalities (Recalcati et al., 2010). In addition to the problems related to the relative importance of each IRP, another complicating factor comes from conflicting results obtained in animal models. Studies in a rat model of chronic cardiotoxicity found no evidence of impaired IRP-1 binding activity following DOX administration (Cusack et al., 2006), but evidence of IRP-1/aconitase sensitivity to DOX was obtained by other authors (Sacco et al., 2003). On the other hand, studies exploiting IRP-1-deficient mice showed that an acute DOX treatment caused cardiac damage irrespective of the presence or absence of IRP-1 (Corna et al., 2006).

Taking into consideration these inconsistencies, one should appreciate the aforesaid pharmacokinetic and metabolic limitations that restrict the validity of the preclinical models of DOX cardiotoxicity. However, it could be concluded that, by allowing ferritin mRNAs translation, an acute loss of IRP-2 might permit free iron sequestration in newly formed ferritin shells before it triggers oxidative damage. This effect would be consistent with the protective effect of ferritin (see below). On the other hand, a chronic loss of IRP-2 is expected to be more injurious, particularly in case IRP-1 is also irreversibly damaged. In fact, the embryonic mortality of IRP-1 and IRP-2 double deficient mice anticipates that complete disruption of the IRP/IRE system is not easily compensated.

### FERRITIN

Another possible mechanism linking iron and anthracycline metabolism involves ferritin. While *in vitro* experiments using purified components suggested that the semiquinone free radical and O_2_^•-^ produced by redox cycling of DOX quinone moiety caused iron release from ferritin (Thomas and Aust, 1986), the role of ferritin *in vivo* seems quite opposite. In fact, recent data suggest that the increase in ferritin synthesis induced by DOX could be a defensive mechanism to limit the amount of iron available for ROS production in the heart, and thus prevent oxidative injury, in line with the known antioxidant function of this protein (Cairo et al., 1995; Arosio and Levi, 2010). We demonstrated that ferritin is induced in H9c2 cardiomyocytes (Corna et al., 2004) and mouse hearts (Corna et al., 2006) exposed to DOX and protected the cells against iron toxicity. Newly formed ferritin can therefore sequester iron and may paradoxically represent a defense for cardiomyocytes. These findings were recently supported by two studies demonstrating that NF-*k*B-mediated ferritin H chain induction plays an important role in the mitochondrial protection offered by metformin against DOX cardiotoxicity in isolated cardiomyocytes (Asensio-López et al., 2013a,b). Moreover, it has been shown that exposure of cardiomyocytes to DOX leads to higher accumulation of iron into ferritin by mechanisms that impair iron release from ferritin and/or lysosomal/proteasomal degradation of this iron-storage protein (Kwok and Richardson, 2003, 2004).

As reported above, a number of observations indicate that cardiac mitochondria are preferential targets of anthracyclines. Additional evidence was recently provided by data showing that the anthracycline epirubicin has an intracellular distribution different from that of DOX: it accumulates in acidic organelles (lysosomes, recycling endosomes) and spares the mitochondria (Salvatorelli et al., 2006). This appears to be one of the reasons for the reduced cardiotoxicity of this analog.

In this context, recent results point to a critical role for mechanisms controlling mitochondrial iron availability in anthracycline cardiotoxicity. In particular, it has been recently shown that exposure to DOX induces iron accumulation specifically in mitochondria, and mice with heart-specific deletion of ABCB8, which is involved in iron export out of the mitochondria, were more sensitive to DOX cardiotoxicity, whereas the opposite effect was obtained with ABCB8 overexpression (Ichikawa et al., 2014). Mitochondrial ferritin (FtMt), which is a recently identified ferritin type that accumulates specifically in the mitochondria where readily accumulates iron (Arosio and Levi, 2010), may be another key player. FtMt expression, which is not controlled by intracellular iron levels, is high in mitochondria-rich tissues, such as the testis, brain, heart. Despite the lack of evident phenotype in FtMt-deficient mice (Bartnikas et al., 2010), studies in cultured cells overexpressing FtMt showed that, by sequestering iron inside the mitochondria, FtMt is able to reduce iron-mediated oxidative damage (Campanella et al., 2009). Moreover, it has been recently demonstrated that ectopic expression of FtMt in HeLa cells was able to reduce DOX-mediated cytotoxicity (Cocco et al., 2013). Although this effect was not obtained in cardiac cells, these results strongly suggested that FtMt may play a relevant role in DOX cardiotoxicity. Indeed, FtMt induction was recently found in the heart of DOX-treated mice (Ichikawa et al., 2014).

Given the role of mitochondria in cellular iron homeostasis, their importance as producers and targets of ROS, and their interaction with anthracyclines, further investigation of the effects of DOX on mitochondrial iron metabolism is required to increase our knowledge of DOX cardiotoxicity.

## CONCLUSIONS AND PERSPECTIVES

Understanding the molecular basis of cardiotoxicity is important to improve the therapeutic use of anthracyclines, which still remain among the most effective antitumor drugs, and to identify less cardiotoxic analogs. In this context, the role of iron in the pathogenesis of cardiac damage is undisputed, but the precise molecular mechanisms underlying its effects remain incompletely understood (**Figure 2**). While early studies pointed to a role as amplifier of the free radical generation initiated by DOX redox cycling, more recent evidence suggests that the cardiotoxic role of iron should not be restricted to the oxidative stress scenario. The fact that anthracycline cardiotoxicity is mitigated by iron chelators but not by antioxidants, together with biochemical evidence showing that proteins important for cellular iron homeostasis are specifically targeted by DOX, indicates that iron and anthracyclines can also induce oxidant-independent cell injury. Exploring more deeply the role of iron in the pathogenic mechanisms of DOX cardiotoxicity will reveal useful insights for the development of improved therapeutic strategies against anthracycline-dependent heart damage and CHF.

## Conflict of Interest Statement

The authors declare that the research was conducted in the absence of any commercial or financial relationships that could be construed as a potential conflict of interest.
